# SPSignal: a web tool for structure-assisted prediction of nuclear localization and nuclear export signals in proteins

**DOI:** 10.1093/nar/gkag421

**Published:** 2026-05-11

**Authors:** Camila Engler, Luciano A Abriata, Nicolas G Bologna

**Affiliations:** Centre for Research in Agricultural Genomics, CSIC-IRTA-UAB-UB, Bellaterra, Barcelona 08193, Spain; Protein Structure Core Facility and Laboratory for Biomolecular Modeling, School of Life Sciences, École Polytechnique Fédérale de Lausanne, CH-1015, Switzerland; Centre for Research in Agricultural Genomics, CSIC-IRTA-UAB-UB, Bellaterra, Barcelona 08193, Spain

## Abstract

Nuclear localization signals (NLSs) and nuclear export signals (NESs) mediate nucleocytoplasmic transport of proteins through the nuclear pore complex and are essential determinants of protein function. However, their short and degenerate sequence patterns frequently lead to high false-positive rates in sequence-based prediction methods, as similar motifs occur widely in proteins without mediating nuclear transport. Here, we present SPSignal, a webserver for improved identification of NLS and NES motifs by integrating sequence-based predictions with structural features. SPSignal combines curated datasets of experimentally validated signals with analyses of solvent accessibility, intrinsic disorder, and structural context derived from experimental or predicted protein structures. Using these features, interpretable machine-learning models based on the RuleFit algorithm prioritize candidate motifs that are structurally exposed and therefore more likely to be functional. The web server integrates sequence predictors with structure-informed analyses in a unified workflow that accepts protein sequences or structures as input and provides interactive visualization of predicted signals within their three-dimensional context. SPSignal assigns confidence scores to candidate motifs and allows users to explore their spatial distribution along protein sequences and structures. Application to proteins with validated localization signals shows that SPSignal improves prediction accuracy by reducing false positives without compromising sensitivity. SPSignal is available at https://sps.cragenomica.es.

## Graphical Abstract

**Graphical Abstract:**
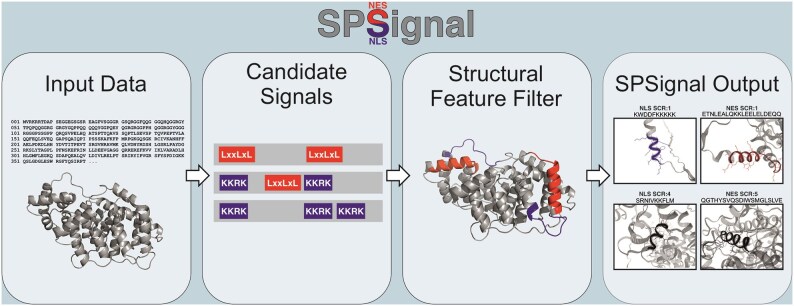

## Introduction

The subcellular localization of proteins is essential for their biological function and influences a wide array of critical cellular processes, including signal transduction, gene expression, and metabolic pathways [1–3]. To function normally, proteins, synthesized in the cytoplasm, are selectively transported into their destination compartments, guided by subcellular localization signals [4–6]. A central role in these transport processes is played by nuclear localization signals (NLSs) and nuclear export signals (NESs), which mediate the active transport of proteins across the nuclear pore complex [5–7]. NLS motifs vary substantially in terms of length and features. However, almost all share a simple feature, short peptide motifs enriched in basic residues, typically positively charged amino acids such as Arginine (R) and Lysine (K), which function as molecular tags directing protein import from the cytoplasm into the nucleus. Classical NLSs are categorized as either monopartite or bipartite, the latter consisting of two clusters of basic residues, both of which are required to determine proper nuclear localization of the protein. Other types of NLS exist, e.g. the Proline(P)-Tyrosine(Y) NLS (R/K/HX_(n)_PY motif, where P denotes Proline, Y Tyrosine, and X any amino acids) [8–10]. Conversely, classical NESs are typically 10–15 amino acids motifs. They consist of four to five hydrophobic residues (Φ), generally Leucine (L), Isoleucine (I), Valine (V), Methionine (M), and Phenylalanine (F). These hydrophobic residues are spaced with various patterns following the consensus Φ^1^-X_(2–3)_-Φ^2^-X_(2–3)_-Φ^3^-X_(1)_-Φ^4^. Subsequent structural studies revealed that NESs may also contain an additional hydrophobic residue (Φ^0^). Based on the pattern of hydrophobic residues and the spacing sequences, NES motifs are classified as class 1a, 1b, 1c, 1d, 2, 3, and 4 [11, 12]. Unlike targeting signals for the endoplasmic reticulum or mitochondria, which are typically short N-terminal peptides that are constitutively exposed and often cleaved after import, NLSs and NESs often remain intact in mature proteins and can be located virtually anywhere in the primary sequence as long as they can become accessible to the import and export machineries in the tertiary structure [4, 5].

Classical NLSs and NESs on cargo proteins are recognized by shuttle proteins known as karyopherins, which include importins and exportins. Classical NLSs on cargo proteins are recognized by the importin α subfamily, which in turn is recognized by the importin β subfamily. The resulting complex between the NLS-containing cargo, importin α, and importin β is then imported into the nucleus [13–15]. Classical NESs are directly recognized by CRM1 (chromosome maintenance protein 1; karyopherin β subfamily), the major nuclear exporter factor, which shuttles between the nucleus and the cytoplasm, releasing its cargo into the cytoplasm. Mechanistic studies on NLS- and NES-containing proteins have shown that these localization signals need to be properly exposed to be effectively recognized by their cognate importins and exportins [16–19]. In some proteins, intramolecular interactions or adjacent regulatory domains can mask a localization signal, making it inaccessible to nuclear transport proteins until conformational changes or specific interactions expose the motif and enable transport, thereby adding a regulatory layer [20].

Despite the advances made in the characterization of NLS and NES signals, the diversity of their sequence, incomplete understanding of consensus patterns, limited training data, and reliance on sequence-only information challenge their prediction accuracy on existing computational methods, often exhibiting high false-positive rates. We and other groups have demonstrated that incorporating tertiary structural information and biochemical features is essential for detecting true NLS and NES motifs, improving the ability to distinguish functional signals [21–25]. With the rapid expansion of structural databases and especially the widespread availability of high-confidence AI-driven protein structure predictions, workflows that integrate sequence information with three-dimensional structural context have now become both feasible and necessary to the accuracy of nuclear transport motif identification. In response to this need, we developed SPSignal, a web tool for Structure-assisted Prediction of NLSs and NESs in proteins that integrates traditional sequence-based predictors with structure-informed analyses, supported by a curated training dataset of biochemical features derived from experimentally validated signals. Based on machine-learning models and interpretable RuleFit scoring built into a single coherent workflow, SPSignal enhances the accuracy of NLS and NES prediction and enables users to evaluate candidate trafficking motifs within their full structural context, providing biologically grounded predictions that are both reproducible and easy to interpret.

## Methods and Results

### Structural characterization of experimentally validated NLS and NES signals

**Figure 1.**
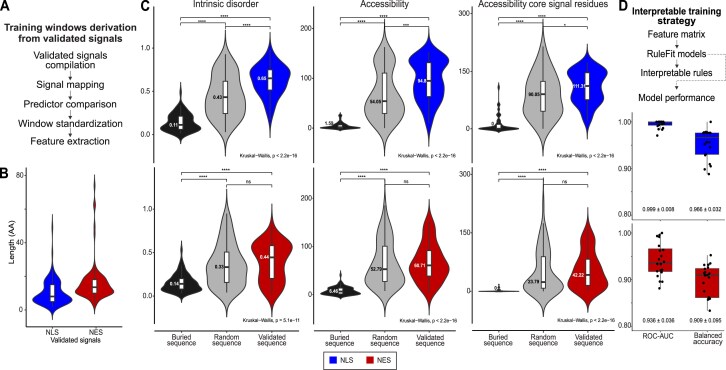
Dataset construction, feature characterization, and model performance. (**A**) Workflow for the derivation of training windows from experimentally validated NLS and NES signals. Validated signals were curated from literature and mapped onto protein sequences, cross-checked with traditional predictors, standardized into fixed-length sequence windows, and used to compute sequence- and structure-derived features. (**B**) Length distribution of experimentally validated NLS (blue) and NES (red) signals used to define standardized sequence windows for model training. (**C**) Structural and biophysical characterization of sequence windows. Violin plots compare intrinsic disorder, solvent accessibility, and accessibility of signal-core residues across buried sequences (negative set), random sequence, and validated signals. Statistical significance was assessed using Kruskal–Wallis tests. (**D**) Interpretable training strategy and model performance. Feature matrices derived from curated datasets were used to train RuleFit models, generating interpretable rules linking structural exposure and sequence properties to signal functionality. Model performance was evaluated using repeated protein-level splits and is shown as receiver operating characteristic area under the curve (ROC-AUC) and balanced accuracy values.

As the reliability of our predictive model depends critically on the quality of the training data, we first constructed a curated dataset of experimentally validated NLS and NES. This process resulted in a high-confidence reference set of 200 experimentally validated NLS and NES (Fig. 1A; Supplementary M&M; Supplementary Table S1). Predictions were further cross-validated using the gold-standard tools NLStradamus [26] and NESmapper [27]. Based on signal-wise distribution profile analysis (Fig. 1B), to ensure compatibility with the fixed-length window-based prediction framework implemented in SPSignal, predicted and experimentally validated regions were mapped to sequence windows of uniform length, of 10 residues for NLS and 20 residues for NES. After window standardization and redundancy filtering, the final dataset comprised 140 proteins containing positive windows corresponding to experimentally validated NLS and NES signals (Supplementary Table S2).

Structural and biophysical features were then systematically computed for each sequence window. Intrinsic disorder was assessed utilizing sequence-based IUPred3 across the entire window [28]. To extract structural features, a model of the three-dimensional structure of the whole protein containing the NLS or NES was retrieved from the AlphaFold [29], UniProt, or NCBI [30] databases when available, or alternatively modeled using LocalColabFold [31]. Mean solvent accessibility across the entire window of each NLS or NES prediction was calculated using NACCESS [32]. In addition, the accessibility of residues mediating interactions with transport receptors was evaluated and averaged across these residues for each signal type (R and K for NLS; F, L, I, M, and V for NES) as shown in comparative Violin Plots (Fig. 1C; Supplementary M&M; Supplementary Table S2) [19, 33]. Altogether, these sequence- and structure-based features provided a comprehensive representation of each candidate signal within its full molecular context, forming the basis for robust model training and evaluation.

### Interpretable rule-based models for NLS and NES prediction

As no curated set of experimentally validated negative localization signals exists, we generated a biologically informed negative pool from the same proteins containing validated signals, thereby avoiding organism- or protein-specific biases. Sliding windows of identical length to positive windows were generated along the full-length sequences, excluding any region overlapping experimentally validated signals; these constituted the random sequences pool. From this pool, a structurally constrained subset termed buried sequences was defined by selecting windows with an average structural depth >3.75 Å, cutoff to distinguish the protein’s buried core from surface layers, based on the rationale that functional NLS and NES motifs must be solvent-accessible to interact with nuclear transport receptors (Fig. 1C and Supplementary Table S3) [16–19, 34, 35]. Importantly, only the buried sequences were used as the negative class for model training, increasing the likelihood that negative examples corresponded to truly non-functional regions.

NLS and NES prediction models were trained independently. The chosen algorithm was RuleFit [36], an interpretable ensemble method that combines decision rules extracted from tree-based models with linear coefficients, characterized by its strong balance between performance and interpretability with a stable generalization. The models were trained as described in Supplementary M&M. To avoid information leakage between training and test sets, model performance was evaluated using repeated train/test splits performed at the protein level. In each split, proteins were randomly assigned to training (70%) or test (30%) sets, ensuring that no protein appeared in both sets. All sequence windows derived from a given protein were assigned consistently to the same split. This procedure was repeated 20 times using different random seeds. For each split, models were trained on the training set and evaluated exclusively on the held-out proteins. Model performance was assessed on test sets using complementary metrics: the ROC-AUC to evaluate overall discriminative ability, and balanced accuracy to account for potential class imbalance and asymmetric error rates. Reported performance values correspond to the mean and standard deviation across repeated protein-level splits (Fig. 1D). Collectively, this framework couples biologically grounded dataset construction with interpretable rule-based learning and stringent protein-level validation, enabling accurate and mechanistically interpretable prediction of NLS and NES signals.

### Implementation of the SPSignal web server

**Figure 2.**
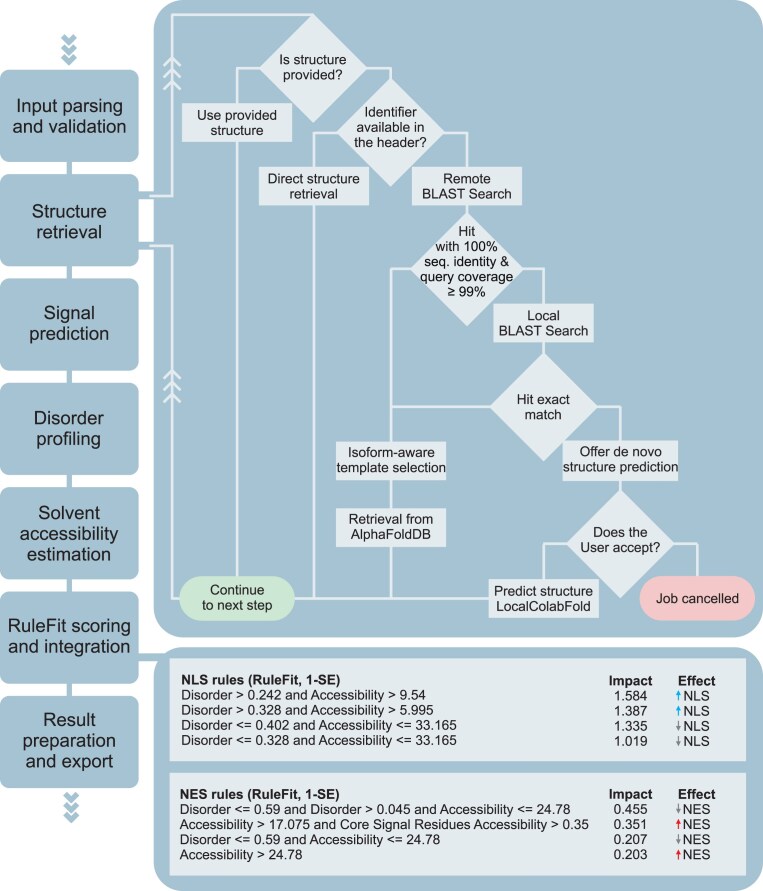
SPSignal computational workflow and interpretable rule-based prediction. Overview of the SPSignal analytical pipeline. The workflow includes input parsing and validation, structure retrieval or prediction, signal detection using sequence-based predictors, structural feature calculation, and final scoring through interpretable machine-learning models. When no structure is provided, SPSignal follows a hierarchical retrieval strategy, prioritizing public structure databases, followed by local sequence searches and, if necessary, *de novo* structure prediction. When a user-provided structure is available, it is used directly and overrides all automated retrieval or prediction steps. The figure also shows representative interpretable rules derived from RuleFit models for NLS and NES prediction, illustrating how combinations of structural features (e.g. intrinsic disorder and solvent accessibility) contribute to classification.

To make this framework accessible to the community, we implemented SPSignal as an interactive web server that integrates sequence- and structure-based analyses within a unified and user-friendly interface. SPSignal combines an interactive web interface with an asynchronous processing backend and an advanced prediction logic, which allows the researchers to analyse and visualize localization signals in proteins in a precise and efficient way. The principal workflow of the tool, illustrating the processing stages from input to results, is shown in the Fig. 2.

The interface provides a dual-input system for protein analysis. Users can either input protein sequences (via manual entry or FASTA-formatted files with .fasta, .txt, or .seq extensions) or upload structural data in PDB/mmCIF formats (Supplementary Fig. S3A). The server currently supports protein sequences of 30–3000 amino acids in length. After input loading and pressing the Start Analysis button, SPSignal starts performing the input parsing, which consists of the validation and sequence normalization (Supplementary Fig. S1B). The second step is structure retrieval, a pipeline that determines the most appropriate 3D model for downstream analysis using a hierarchical, fallback-based strategy. If the user does not provide a structure, the system evaluates the availability of a protein identifier in the sequence entry to facilitate direct retrieval from established repositories. When an identifier is absent, the workflow initiates a remote BLAST search against UniProt and NCBI databases. If this search yields a high-confidence hit, characterized by 100% sequence identity and a query coverage of at least 99%, the corresponding structure is retrieved. Alternatively, if these criteria are not met, a local UniProtKB BLAST search is performed to identify potential matches. An exact match at this stage triggers an isoform-aware template selection process, followed by structural retrieval from the AlphaFold Database. In the absence of an exact match, the system prompts the user with the option of *de novo* prediction. If accepted, the protein structure is computationally modeled using AlphaFold 2 via LocalColabFold; otherwise, the task is terminated (Fig. 2). When a structure is explicitly provided by the user, it is used directly and overrides any automatically retrieved or predicted models. Ultimately, all successful acquisition paths converge to proceed with the subsequent stages of the analytical pipeline. Further implementation details, including typical runtimes and queue behavior, are described in the Supplementary Materials and Methods.

The workflow continues with the identification and processing of putative NLS and NES motifs using NLStradamus and NESmapper, and for each of these candidate signals, the intrinsic disorder and the estimation of solvent accessibility, including both window-level and residue-specific values relevant to each signal are calculated. While the current model does not explicitly label NLS as monopartite or bipartite, the users can identify both signal types (See Supplementary Materials and Methods for details). Once the feature extraction is complete, this information is formatted into a matrix and fed into the corresponding RuleFit model to compute prediction scores which classify every signal and assess confidence (Supplementary Table S4). Each candidate NLS or NES is assigned a probability-based SPSignal Confidence Rank (SCR) ranging from 1 (highest confidence of being a structurally exposed functional signal) to 5 (lowest confidence). During execution, a synchronized progress widget offers real-time monitoring, displaying both the percentage of completion and the current task. For easy tracking, the active Job ID appears right below this bar (Supplementary Fig. S1B), allowing posterior retrieval.

**Figure 3.**
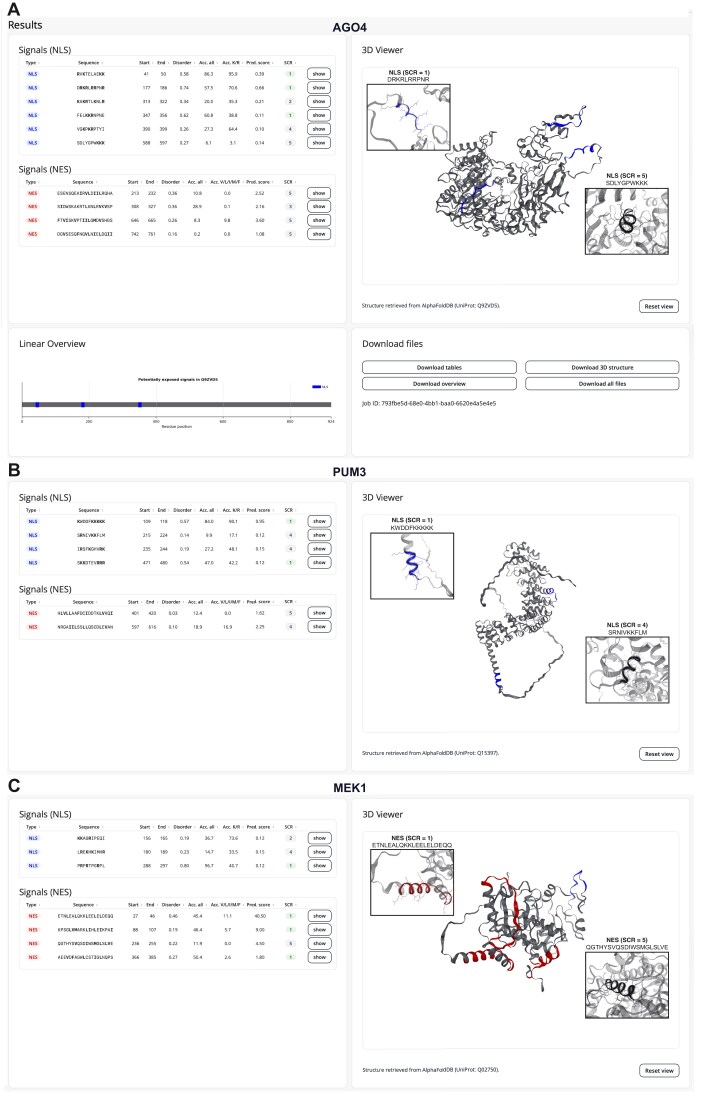
SPSignal output and visualization of predicted localization signals. (**A**) Output interface displaying predicted NLS and NES signals together with their calculated features, prediction scores, and SCR in interactive tables. Predicted signals can be visualized interactively in a three-dimensional protein structure viewer, enabling exploration of their structural exposure. Linear sequence representations allow users to inspect the distribution of candidate signals along the protein sequence. A separate panel provides access to result tables, structural files, and summary outputs for download. A unique Job ID is assigned to the analysis for retrieval and reference. The example shown corresponds to the AGO4 protein analyzed in the case studies (UniProt: Q9ZVD5; Job ID: dd26dab2-6941-44f3-8f5e-4e9a0e028d7f). Insets illustrate examples of signals with high confidence (SCR = 1) and low confidence (SCR = 5) based on structural accessibility. Representative analyses of human proteins PUM3 (**B**) (UniProt: Q15397; Job ID: 3d339f73-8a4a-4487-a31f-dda4e0d83fab), and MEK1 (**C**) (UniProt: Q02750; Job ID: 0ec9b415-4b5e-4d74-a465-2308f6fa7950). Insets illustrate examples of high- and low-confidence signals mapped onto the three-dimensional structures.

Once the analysis is complete, the final output is dynamically rendered at the bottom of the page. Outputs include dynamic tables with the details on each signal analyzed (Sequence, Position, Calculated properties, Prediction Score, and SCR) (Fig. 3A). While SCR1 signals are typically clearly exposed and likely functional, and SCR5 signals are largely buried and unlikely to be functional, we recommend careful manual inspection of intermediate cases (SCR2–SCR4), particularly those in SCR2. In addition, individual interactive buttons provide visualization of the corresponding color-coded signals within their structural context, allowing users to freely rotate, pan, and zoom the structure using mouse or touch gestures for detailed inspection of specific regions. A linear representation of the protein sequence is also displayed, allowing users to quickly visualize the location and distribution of predicted NLS and NES motifs along the full-length protein. Dedicated buttons allow users to initiate the analysis, run an example, or cancel a current job. The “Load Previous Analysis” feature provides a practical shortcut: by entering a saved Job ID, results can be retrieved instantly without re-running the process or staying online (Supplementary Fig. S1B). Job results are kept in the server for at least 1 week, with the possibility of longer storage depending on server usage and resource availability. In addition, SPSignal allows users to download all generated outputs, either individually or as a single compressed package (Fig. 3A). Complementing the main dashboard, menu panel provides access to supplementary information, including a comprehensive User Manual with detailed documentation covering everything from input definitions and parameter logic to output interpretation and troubleshooting guides to ensure a seamless analytical workflow. SPSignal is freely accessible online without registration and does not require any email input to obtain results and deployed on an HTTPS-secured server with Celery/Redis-based job management.

### Case study: SPSignal analysis of proteins with validated localization signals

To illustrate the practical application of SPSignal, we analyzed proteins containing experimentally validated NLS or NES that were not included in the training or testing dataset used for model development. These examples demonstrate how SPSignal integrates sequence-based predictions with structural context to prioritize functional motifs.

#### Case study 1: identification of a functional NLS within the globular domain of Arabidopsis ARGONAUTE4

As a first example, the ARGONAUTE4 protein (AGO4) from *Arabidopsis thaliana* was examined using SPSignal. AGO4 plays a central role in RNA-directed DNA methylation and accumulates predominantly in the nucleus [37]. Previous studies identified a functional NLS located within the AGO4 protein essential for AGO4 nuclear localization [38, 39]. Sequence-based predictors detect six potential NLS candidates (Fig. 3A). SPSignal evaluates these predictions using structural and biophysical features, assigning high-confidence ranks (SCR = 1) to three candidate motifs, whereas the remaining motifs receive lower confidence ranks due to limited structural accessibility. Among the three motifs prioritized by SPSignal, the experimentally validated NLS (DRKRLRRPNR) corresponds to a solvent-exposed loop within the structured MID domain. The other two predicted candidates would in this scenario require experimental validation. On the side of NES, sequence-based analysis identifies four potential motifs within AGO4; however, SPSignal assigns low confidence to all these candidates, as structural analysis reveals that the hydrophobic residues forming these motifs are buried within the compact AGO4 fold (Fig. 3A). Consistent with this prediction, no functional NES has been experimentally validated for AGO4 to date.

#### Case study 2: identification of a functional NLS in the human PUM3 protein

As a second example, the human protein Pumilio homolog 3 (PUM3) was examined using SPSignal (Fig. 3B). PUM3, also known as PUF-A, is a nucleolar RNA- and DNA-binding protein implicated in ribosome biogenesis and cellular stress responses. PUM3 participates in nucleolar ribosomal RNA processing and ribosomal assembly and has been linked to regulation of the DNA damage response and tumor cell proliferation [40–42]. A classical NLS located between residues 106 and 118 has been experimentally validated in this protein [40]. Sequence-based predictors identify four basic residue clusters across the PUM3 sequence as potential NLS candidates. Based on structural accessibility and disorder, SPSignal prioritizes two candidate motifs with a Confidence Rank of SCR = 1, including the experimentally validated NLS, due to its high solvent accessibility and elevated intrinsic disorder. Regarding NES predictions, two candidate motifs are assigned low-confidence scores (SCR = 4 and 5) by SPSignal, as structural analysis indicates that the hydrophobic residues forming these motifs are buried within the protein structure and therefore unlikely to be accessible for interaction with the nuclear export receptor CRM1 (Fig. 3B).

#### Case study 3: identification of a functional NES in the human MEK1 protein

As a third example, the human protein Mitogen-activated protein kinase kinase 1, MEK1, was analyzed using SPSignal (Fig. 3C). MEK1 is a key component of the MAPK signaling pathway, shuttling between the nucleus and cytoplasm to regulate downstream transcriptional responses [43–45]. A functional NES located in the N-terminal region of MEK1 has been experimentally validated to mediate CRM1-dependent nuclear export [46–48]. Sequence-based predictors identify four motifs across the MEK1 sequence as potential NES candidates. SPSignal analysis assigns high-confidence scores to three motifs based on structural accessibility and disorder criteria, including the experimentally validated NES (27–46 amino acids). Regarding nuclear import, although MEK1 can transiently accumulate in the nucleus upon mitogenic stimulation, no canonical NLS has been experimentally validated for this protein. Sequence-based predictors identify three clusters of basic residues in the MEK1 sequence as potential NLS motifs. SPSignal assigns a high-confidence score (SCR = 1) to only one of these candidates based on structural accessibility, whereas the others receive lower confidence ranks (SCR = 4 and 5) and correspond to structurally buried motifs unlikely to function as NLS (Fig. 3C).

#### Case study 4: identification of a functional NES and NLS in the human UPF2 protein

As a fourth example, the human protein Up-frameshift protein 2 (UPF2) was analyzed using SPSignal (Supplementary Fig. S1C). UPF2 is a core component of the nonsense-mediated messenger RNA (mRNA) decay pathway and functions together with UPF1 and UPF3 to recognize and degrade aberrant mRNAs [49, 50]. Consistent with its role in RNA surveillance, UPF2 associates with ribonucleoprotein complexes involved in nucleocytoplasmic RNA metabolism. UPF2 represents a particularly informative example because it is a large multidomain protein of >1200 amino acids. In such long proteins, the degenerate nature of NLS and NES consensus motifs frequently generates numerous sequence matches that are unlikely to be functional. Sequence-based predictors identify multiple hydrophobic sequence motifs across the UPF2 sequence as potential NES candidates. In total, 12 motifs match consensus patterns typical of leucine-rich NES. SPSignal evaluates these candidates using structural accessibility features, assigning high-confidence scores (SCR = 1) to six motifs, among which is the experimentally validated NES located between residues 512 and 531 [51]. Regarding NLS predictions, sequence-based methods identify 10 clusters enriched in basic residues across the UPF2 sequence as potential NLS motifs. SPSignal assigns high-confidence scores to six motifs based on structural accessibility and disorder criteria. Previous studies reported that two fragments from the N-terminal region of UPF2 are sufficient to direct nuclear localization of a reporter protein [52, 53]. Based on structural accessibility and disorder, SPSignal prioritizes two candidate motifs (KRKKEDKERK and LKKNTAFVKK) with a Confidence Rank of SCR = 1, which overlap with these two regions, suggesting that they may represent the functional NLS elements responsible for nuclear targeting (Supplementary Fig. S1C).

In summary, these case studies, together with additional examples provided in the online manual, illustrate how SPSignal prioritizes structurally accessible and biologically plausible nuclear trafficking signals while reducing spurious sequence-based predictions. In total, we analyzed 31 cases (NLS 39%; NES 61%) from different species (animals 71%; plants 29%) that were included neither in the training nor testing datasets used for model development. Sequence-based prediction methods alone identified 263 candidate NLS/NES motifs. After applying SPSignal, the number of candidate signals was reduced by 35%–40%. Importantly, experimentally validated signals were retained with high sensitivity (100%), resulting in a strong enrichment of true positives relative to the total number of predictions (Supplementary Fig. S1D and Supplementary Table S5). This effect was more pronounced in proteins with lower intrinsic disorder and bigger size, as expected. Together, these results highlight the ability of SPSignal to improve the specificity of NLS/NES prediction without compromising sensitivity, while simplifying analysis by eliminating multi-step manual workflows, leading to reduced complexity and analysis time.

## Conclusion

Accurate identification of NLS and NES remains a challenging problem due to the short and degenerate nature of these motifs. SPSignal addresses this limitation by integrating sequence-based predictions with structural and biophysical features that reflect the molecular context in which these signals operate. Subcellular transport receptors recognize motifs that are exposed on the protein surface and accessible for interaction. Therefore, incorporating information on solvent accessibility, intrinsic disorder, and structural depth provides a biologically meaningful framework to distinguish functional signals from buried or structurally constrained sequence matches. The results presented here demonstrate that combining curated training datasets with structure-informed features and interpretable machine-learning models improves the prioritization of candidate NLS and NES motifs while maintaining transparency in the decision-making process. Across a diverse set of proteins with experimentally validated signals, SPSignal improves prediction accuracy by reducing false positives without compromising sensitivity. This improvement is observed in direct comparison with sequence-based predictors, highlighting the added value of SPSignal as a structure-informed post-processing framework. These results highlight the potential of SPSignal to guide experimental design by narrowing down candidate motifs to those most likely to be functional. Despite SPSignal's advances, several limitations remain. The number of experimentally validated NLS and NES signals are still scarce relative to the vast diversity of proteins across eukaryotes. In addition, the current use of structurally buried regions as negative examples represents a conservative strategy, constrained by the lack of experimentally validated negative datasets, and may underestimate the complexity of distinguishing functional from non-functional surface-adjacent motifs. Expanding curated datasets with additional experimentally validated positive and negative signals will further improve model performance and generalizability. We further encourage the community to share newly experimentally validated NLS and NES motifs, including negative results, which will support ongoing refinement of SPSignal and improve future versions of the method. Future developments may also incorporate additional structural descriptors, dynamic conformational information, or protein-protein interaction contexts that influence signal accessibility. In addition, although the current implementation integrates the widely used predictors NLStradamus and NESmapper for initial motif detection, the modular architecture of SPSignal allows straightforward integration of additional NLS and NES predictors [11, 54–59]. More broadly, the conceptual framework implemented in SPSignal could be extended to other classes of short functional sequence elements, including post-translational modification sites, degrons, or protein-protein interaction motifs, where integrating sequence patterns with structural exposure and biophysical context may similarly improve the identification of functional regulatory regions [60–64].

## Supplementary Material

gkag421_Supplemental_Files

## Data Availability

SPSignal is free and open to all users, and there is no login requirement. It’s available at https://sps.cragenomica.es Source code is available at https://doi.org/10.5281/zenodo.19663416.
