# Supplementary material for: SPSignal: a web tool for structure-assisted prediction of nuclear localization and nuclear export signals in proteins
Source: Nucleic Acids Res. 2026 May 11;54(W1):W327–36. doi: 10.1093/nar/gkag421 (PMC13355057; doi:10.1093/nar/gkag421)
Supplement: gkag421_Supplemental_Files [file gkag421_supplemental_files.zip › Engler et al_SPSignal_Supp Figures_Revision.pdf]

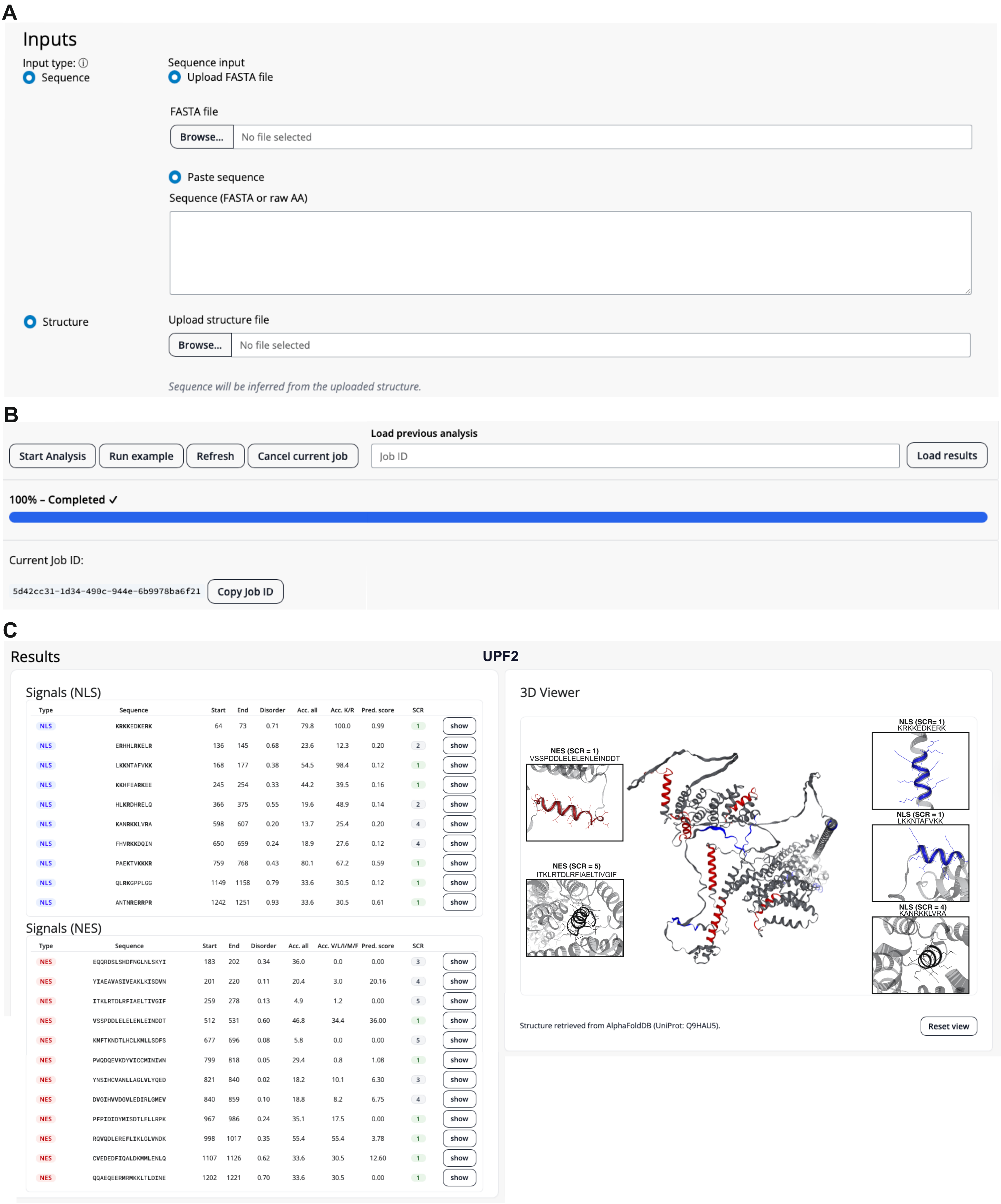

Signals (NLS)

Type	Sequence	Start	End	Disorder	Acc. all	Acc. K/R	Pred. score	SCR	
NLS	KRKKEDKERK	64	73	0.71	79.8	100.0	0.99	1	show
NLS	ERHHLRKELR	136	145	0.68	23.6	12.3	0.20	2	show
NLS	LKKNTAFVKK	168	177	0.38	54.5	98.4	0.12	1	show
NLS	KKHFEARKEE	245	254	0.33	44.2	39.5	0.16	1	show
NLS	HLKRDHRELQ	366	375	0.55	19.6	48.9	0.14	2	show
NLS	KANRKKLVRA	598	607	0.20	13.7	25.4	0.20	4	show
NLS	FHVRKKDQIN	650	659	0.24	18.9	27.6	0.12	4	show
NLS	PAEKTVKKKR	759	768	0.43	80.1	67.2	0.59	1	show
NLS	QLRKGPPLGG	1149	1158	0.79	33.6	30.5	0.12	1	show
NLS	ANTNRERRPR	1242	1251	0.93	33.6	30.5	0.61	1	show

Signals (NES)

Type	Sequence	Start	End	Disorder	Acc. all	Acc. V/L/I/M/F	Pred. score	SCR	
NES	EQQRDSLSHDFNGLNLSKYI	183	202	0.34	36.0	0.0	0.00	3	show
NES	YIAEAVASIVEAKLKISDVN	201	220	0.11	20.4	3.0	20.16	4	show
NES	ITKLRTDLRFIAELTIVGIF	259	278	0.13	4.9	1.2	0.00	5	show
NES	VSSPDDLELELENLEINDDT	512	531	0.60	46.8	34.4	36.00	1	show
NES	KMFTKNDTLHCLKMLLSDFS	677	696	0.08	5.8	0.0	0.00	5	show
NES	PWQDQEVKDYVICCMINIWN	799	818	0.05	29.4	0.8	1.08	1	show
NES	YNSIHCVANLLAGLVLYQED	821	840	0.02	18.2	10.1	6.30	3	show
NES	DVGIHVVDGVLEDIRLGMEV	840	859	0.10	18.8	8.2	6.75	4	show
NES	PFPIDIDYMISDTLELLRPK	967	986	0.24	35.1	17.5	0.00	1	show
NES	RQVQDLEREFLIKLGLVNDK	998	1017	0.35	55.4	55.4	3.78	1	show
NES	CVEDEDFIQALDKMMLENLQ	1107	1126	0.62	33.6	30.5	12.60	1	show
NES	QQAEQEERMRMKKLTLDINE	1202	1221	0.70	33.6	30.5	0.00	1	show

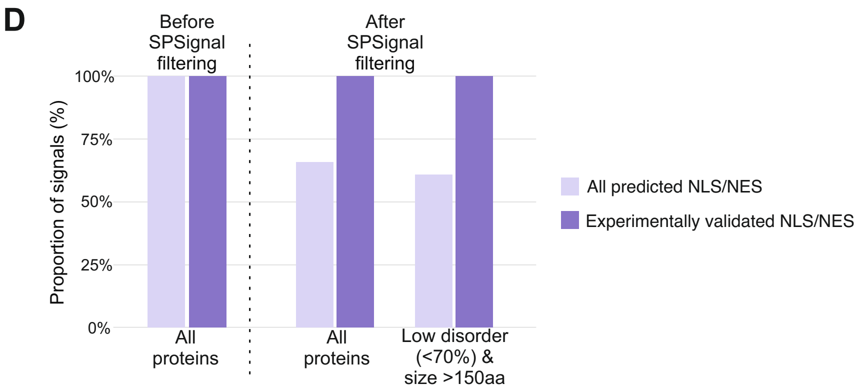

Supplementary Figure S1
