# Supplementary material for: SPSignal: a web tool for structure-assisted prediction of nuclear localization and nuclear export signals in proteins
Source: Nucleic Acids Res. 2026 May 11;54(W1):W327–36. doi: 10.1093/nar/gkag421 (PMC13355057; doi:10.1093/nar/gkag421)
Supplement: gkag421_Supplemental_Files [file gkag421_supplemental_files.zip › Engler et al_SPSignal_SuppM&M_Clean.pdf]

SUPPLEMENTARY MATERIALS & METHODS

Dataset construction

Experimentally validated NLS and NES were compiled through automated literature mining followed by extensive manual curation. An initial corpus of over 1,000 scientific articles describing NLS and NES were analysed, retaining only studies providing direct experimental evidence of signal functionality. Extracted annotations were cross-referenced with external databases including UniProt (1), NESdb (2), and LocNES (3) to verify sequence accuracy and annotation consistency. The curated dataset initially contained 100 validated NLS and 100 validated NES. Additional manual filtering ensured broad taxonomic representation while avoiding overrepresentation of individual protein families or model organisms. Each signal was further evaluated using classical predictive algorithms: NESmapper (4) for NES (minimum score 0) and NLStradamus (5) for NLS (model 2, threshold 0.25) to assess overlap with existing prediction methods. NLStradamus allows selection between a 2-state model and a 4-state model. The 2-state model captures simple signal versus non-signal patterns (typically corresponding to monopartite signals), whereas the 4-state model accounts for more complex architectures by modeling two basic clusters separated by a linker region, thereby enabling detection of both monopartite and bipartite NLSs. To standardize signal length for downstream analysis, a windowing strategy was applied based on the typical length distribution of experimentally validated signals. For each validated instance, the overlap between the experimentally annotated region and predicted signal regions was evaluated to define a representative window centred on the validated signal. When necessary, signal boundaries were extended along the predicted direction to ensure a consistent window size while preserving maximal overlap with the experimentally supported region. Signals shorter than three amino acids were excluded to avoid poorly defined or biologically ambiguous cases. Negative control windows were derived from the same proteins containing experimentally validated signals. Sliding windows of 10 residues (NLS) and 20 residues (NES) were generated across each protein sequence and filtered to exclude regions overlapping validated signals or positive windows (Random Sequences pool). Windows were further filtered by structural accessibility, retaining only those with an average residue depth greater than 3.75 Å (Buried Sequences), reflecting the requirement that functional motifs remain solvent-accessible to interact with nuclear transport receptors. The final negative dataset was balanced with the positive class, resulting in 70 negative windows per signal type.

Feature extraction

Structural and biophysical features were computed for each sequence window. Intrinsic disorder was estimated using IUPred3 (long disorder model) (7), calculating the geometric mean disorder score across each window. Solvent accessibility for whole motifs was obtained using NACCESS (8) to estimate relative solvent accessibility (RSA) with a probe radius of 5.0 Å. This probe approximates the size of a large amino acid side chain and was found to yield better results than the traditionally used water probe radius or probe radii representing carrier protein size. Additionally, accessibility of key residues was evaluated for each signal type. For NLS, only basic residues (R and K) were considered, as X-ray structures of NLS-carrier complexes show the positively charged residues are the main interactors (9). Meanwhile, for NES the analysis focused on hydrophobic residues (F, L, I, M and V), which are found in X-ray structures to be the main interactors with CRM1 (10). Feature values were computed independently for each window without incorporating information from adjacent regions. Feature distributions were compared against random windows and buried regions. Statistical

significance was assessed using the Kruskal-Wallis test followed by pairwise Wilcoxon rank-sum tests. P-values < 0.05 were considered statistically significant.

Prediction model

Independent models were trained for NLS and NES prediction using the RuleFit algorithm, which extracts interpretable decision rules from ensemble tree models and combines them through regularized regression (11). Two base learners were explored: Random Forest (300 estimators, tree depth 1-5) and Gradient Boosting (200 estimators, depth 2-4). To ensure interpretability for integration into the web tool, the number of extracted rules was constrained between 5 and 20. Model hyperparameters, including the maximum number of rules, were optimized using stratified cross-validation on the training data. To select parsimonious yet robust models, we applied the 1-standard-error (1-SE) criterion, choosing the simplest model whose performance was within one standard deviation of the best-performing configuration. The evaluation was conducted using a nested cross-validation scheme based on protein identity. The outer loop consisted of 20 GroupShuffleSplit iterations for test set allocation, while the inner loop employed 5-fold GroupKFold for model selection and hyperparameter tuning. Final performance was summarized across outer splits using the median and standard deviation for AUC-ROC, Balanced Accuracy, Matthews Correlation Coefficient (MCC), and Precision–Recall AUC. Stability of extracted rules was also evaluated across cross-validation partitions.

The SPSignal Confidence Rank (SCR) was defined based on the distribution of RuleFit model output scores across experimentally validated signals. The SPSignal Confidence Rank (SCR) was defined based on the distribution of RuleFit model output scores for experimentally validated signals. The threshold for SCR1 was set at the score corresponding to the 85th percentile of validated signals, ensuring high sensitivity for top-ranked predictions. The threshold for SCR5 was defined symmetrically with respect to the score range, using the same distance from the lower bound (0th percentile) as SCR1 from the upper bound (100th percentile). The intermediate ranks (SCR2–SCR4) were assigned by evenly partitioning the score interval between the SCR1 and SCR5 thresholds.

Implementation details and computational performance

SPSignal is implemented as a multi-stage computational pipeline that integrates sequence-based prediction with structure-aware analysis. Following input validation, the system retrieves or generates a three-dimensional protein structure using a hierarchical strategy: (i) direct use of user-provided structures, (ii) retrieval from public structure databases (e.g., AlphaFoldDB, ESMAtlas, SWISS-MODEL; typically 3-15 min), (iii) fallback searches against a local UniProtKB BLAST database (15-45 min), and (iv) de novo structure prediction using local-colabfold as a final option, which may require several hours depending on sequence length. Once a structure is obtained, SPSignal performs NLS and NES prediction (NLStradamus and NESmapper), intrinsic disorder estimation (IUPred3), solvent accessibility calculation (NACCESS), and feature integration, followed by classification using RuleFit models each requiring less than a few minutes (see manual for detailed information). The server accepts single protein sequences of 30-3000 amino acids composed exclusively of standard residues. Execution time may increase depending on sequence length and the need for structure prediction. To optimize performance and minimize user waiting time, SPSignal incorporates a queue management system with prioritized execution of structure retrieval tasks over de novo prediction. In addition, separate queuing streams are implemented to ensure that structure search and template-based analyses remain responsive and are not delayed by computationally intensive prediction jobs. This design enables efficient handling of

heterogeneous workloads while maintaining rapid turnaround for the majority of user submissions. For most commonly studied proteins, including wild-type sequences, suitable structural models are typically available from public databases. However, in cases involving proteins from less-represented species, rare isoforms, or engineered variants, de novo structure prediction may be required as a final step in the pipeline.

REFERENCES

1. The UniProt Consortium. UniProt: a worldwide hub of protein knowledge. *Nucleic Acids Res* 2019;**47**(D1):D506–15. 10.1093/nar/gky1049.

2. Xu D, Farmer A, Collett G *et al. Sequence and Structural Analyses of Nuclear Export Signals in the NESdb Database | Molecular Biology of the Cell*. 2012;**23**(18):3677–93. 10.1091/mbc.e12-01-0046.

3. Xu D, Marquis K, Pei J *et al.* LocNES: a computational tool for locating classical NESs in CRM1 cargo proteins. *Bioinformatics* 2015;**31**(9):1357–65. 10.1093/bioinformatics/btu826.

4. Kosugi S, Yanagawa H, Terauchi R *et al.* NESmapper: Accurate Prediction of Leucine-Rich Nuclear Export Signals Using Activity-Based Profiles. *PLoS Comput Biol* 2014;**10**(9):e1003841. 10.1371/journal.pcbi.1003841.

5. Nguyen Ba AN, Pogoutse A, Provart N *et al.* NLStradamus: a simple Hidden Markov Model for nuclear localization signal prediction. *BMC Bioinformatics* 2009;**10**(1):202. 10.1186/1471-2105-10-202.

6. Tan KP, Nguyen TB, Patel S *et al.* Depth: a web server to compute depth, cavity sizes, detect potential small-molecule ligand-binding cavities and predict the pKa of ionizable residues in proteins. *Nucleic Acids Res* 2013;**41**(W1):W314–21. 10.1093/nar/gkt503.

7. Erdős G, Pajkos M, Dosztányi Z. IUPred3: prediction of protein disorder enhanced with unambiguous experimental annotation and visualization of evolutionary conservation. *Nucleic Acids Research* 2021;**49**(W1):W297–303. 10.1093/nar/gkab408.

8. Hubbard SJ. *NACCESS-Computer Program*. 1993. https://cir.nii.ac.jp/crid/1370004237630036229 (19 Dec. 2023, date last accessed).

9. Conti E, Uy M, Leighton L *et al.* Crystallographic Analysis of the Recognition of a Nuclear Localization Signal by the Nuclear Import Factor Karyopherin α. *Cell* 1998;**94**(2):193–204. 10.1016/S0092-8674(00)81419-1.

10. Dong X, Biswas A, Süel KE *et al.* Structural basis for leucine-rich nuclear export signal recognition by CRM1. *Nature* 2009;**458**(7242):1136–41. 10.1038/nature07975.

11. Friedman JH, Popescu BE. Predictive learning via rule ensembles. *Ann Appl Stat* 2008;**2**(3). 10.1214/07-AOAS148.

SUPPLEMENTARY FIGURE AND TABLE LEGENDS

Supplementary Figure S1. SPSignal web interface and visualization of predicted localization signals. (A) Input interface of the SPSignal web server. Users can submit protein sequences in FASTA format, raw text, or upload structural files (PDB/mmCIF) for analysis. (B) Job monitoring panel showing real-time execution progress and job identification for tracking or retrieving previous analyses. (C) Representative analysis of human protein UPF2 (UniProt: Q9HAU5; Job ID: c5cbfdb2-13a1-4103-a747-10d74a978009). Insets illustrate examples of high- and low-confidence signals mapped onto the three-dimensional structures. (D) Comparison of candidate NLS/NES predictions before and after SPSignal filtering. The increase in the proportion of experimentally validated signals relative to the total number of predicted signals is more pronounced in proteins with lower intrinsic disorder (<70% of residues with intrinsic disorder >0.5) and larger size (>150 aa).

SUPPLEMENTARY TABLE LEGENDS

Supplementary Table S1: Curated dataset of experimentally validated NLS and NES.

Supplementary Table S2: Standardized sequence windows and computed structural and biophysical features for experimentally validated NLS and NES.

Supplementary Table S3: Random and structurally buried sequence windows used as negative datasets for SPSignal model training.

Supplementary Table S4: Representative RuleFit rules for candidate NLS and NES motifs.

Supplementary Table S5: List of proteins analyzed in the case studies, including sequence information and annotated and SPSignal-predicted NLS and NES motifs.
